# Metabolic Reprogramming and Risk Stratification of Hepatocellular Carcinoma Studied by Using Gas Chromatography–Mass Spectrometry-Based Metabolomics

**DOI:** 10.3390/cancers14010231

**Published:** 2022-01-04

**Authors:** Chengnan Fang, Hui Wang, Zhikun Lin, Xinyu Liu, Liwei Dong, Tianyi Jiang, Yexiong Tan, Zhen Ning, Yaorui Ye, Guang Tan, Guowang Xu

**Affiliations:** 1 CAS Key Laboratory of Separation Science for Analytical Chemistry, Dalian Institute of Chemical Physics, Chinese Academy of Sciences, Dalian 116023, China; chengnanf@dicp.ac.cn (C.F.); liuxy2012@dicp.ac.cn (X.L.); g1808@dicp.ac.cn (Y.Y.); 2 University of Chinese Academy of Sciences, Beijing 100049, China; 3 International Cooperation Laboratory on Signal Transduction, Eastern Hepatobiliary Surgery Institute, The Second Military Medical University, Shanghai 200438, China; 13816444416@163.com (H.W.); dlw@smmu.edu.cn (L.D.); 08300700057@fudan.edu.cn (T.J.); yxtan1214@163.com (Y.T.); 4 Department of Hepatobiliary Surgery, The First Affiliated Hospital of Dalian Medical University, Dalian 116011, China; linzk@dicp.ac.cn (Z.L.); ningzhen@dmu.edu.cn (Z.N.)

**Keywords:** hepatocellular carcinoma, prognosis, metabolomics, risk stratification, nonnegative matrix factorization

## Abstract

**Simple Summary:**

Hepatocellular carcinoma (HCC) displays dismal prognosis even after surgical resection. Metabolic reprogramming is a hallmark of cancers, but the existence of tumor heterogeneity makes it difficult to comprehensively reflect the overall characteristics of HCC prognosis with only a single or a few biomarkers. The aim of our study was to elucidate HCC metabolic reprogramming based on metabolomics and enable HCC prognostic risk evaluation using metabolic characteristics. We identified three distinct metabolic clusters and a metabolite classifier composed of six fatty acids for HCC prognosis risk stratification, which was externally validated in another independent dataset. Metabolic classification may provide a new insight into the molecular pathological characteristics of HCC for clinical prognosis evaluation and personalized treatment.

**Abstract:**

Hepatocellular carcinoma (HCC) displays a high degree of metabolic and phenotypic heterogeneity and has dismal prognosis in most patients. Here, a gas chromatography–mass spectrometry (GC-MS)-based nontargeted metabolomics method was applied to analyze the metabolic profiling of 130 pairs of hepatocellular tumor tissues and matched adjacent noncancerous tissues from HCC patients. A total of 81 differential metabolites were identified by paired nonparametric test with false discovery rate correction to compare tumor tissues with adjacent noncancerous tissues. Results demonstrated that the metabolic reprogramming of HCC was mainly characterized by highly active glycolysis, enhanced fatty acid metabolism and inhibited tricarboxylic acid cycle, which satisfied the energy and biomass demands for tumor initiation and progression, meanwhile reducing apoptosis by counteracting oxidative stress. Risk stratification was performed based on the differential metabolites between tumor and adjacent noncancerous tissues by using nonnegative matrix factorization clustering. Three metabolic clusters displaying different characteristics were identified, and the cluster with higher levels of free fatty acids (FFAs) in tumors showed a worse prognosis. Finally, a metabolite classifier composed of six FFAs was further verified in a dependent sample set to have potential to define the patients with poor prognosis. Together, our results offered insights into the molecular pathological characteristics of HCC.

## 1. Introduction

Liver cancer, one of the most common malignances, accounts for about 8.4 million new cases and 7.8 million deaths annually worldwide [1], and hepatocellular carcinoma (HCC), as an aggressive cancer with dismal prognosis, accounting for around 90% of primary liver cancers, typically appears in patients with cirrhosis and chronic infection [2]. Owing to death from recurrence, metastasis or the underlying liver disease even after surgical resection, which affects the surgical efficacy and survival of patients seriously, the long-term survival of HCC is not satisfactory [3]. Different organizations have summarized several clinical staging systems to define prognostic subclasses and personalized therapies [4,5,6,7]; however, there is still no consensus on the optimal classification system, which influences the therapeutic effect and survival of HCC [8,9].

Metabolic reprogramming is a hallmark of cancers and participates directly in the tumorigenesis [10,11]. Metabolic disorder affects the biological behavior of solid tissues, and the reprogrammed metabolism of tissues could better reflect the functional state of an organ. Metabolomics study based on tissue is a useful strategy for studying the metabolic abnormalities of diseases, which could provide information about the metabolic modifications and regulatory mechanism, further helping to clarify the basic cancer pathophysiology and provide potential therapeutic targets for clinical treatment [12]. Currently, metabolomics studies of HCC tissues are mainly conducted to characterize differential metabolic features in cancers and further exploited to identify early diagnostic and prognostic markers and understand the pathogenesis mechanism [13,14,15].

Different genotypes or phenotypes existing in the same tumor due to their heterogeneity result in difference in cell growth, drug sensitivity and prognosis. Although a variety of prognostic biomarkers of HCC have been reported by previous studies [5,16,17], it is difficult to comprehensively reflect the overall characteristics of prognosis with only a single or a few biomarkers. As shown in previous research, an expression pattern of gene tags screened from the vast tumor genome to enable cancer classification could reflect tumor status more comprehensively than a single gene and also be more conducive to predict the prognosis of patients [18]. Similarly, it is worthy trying to identify the HCC metabotype related to prognosis based on a pattern of tissue metabolic characteristics, which may contribute to personalized healthcare.

**Figure 1:**
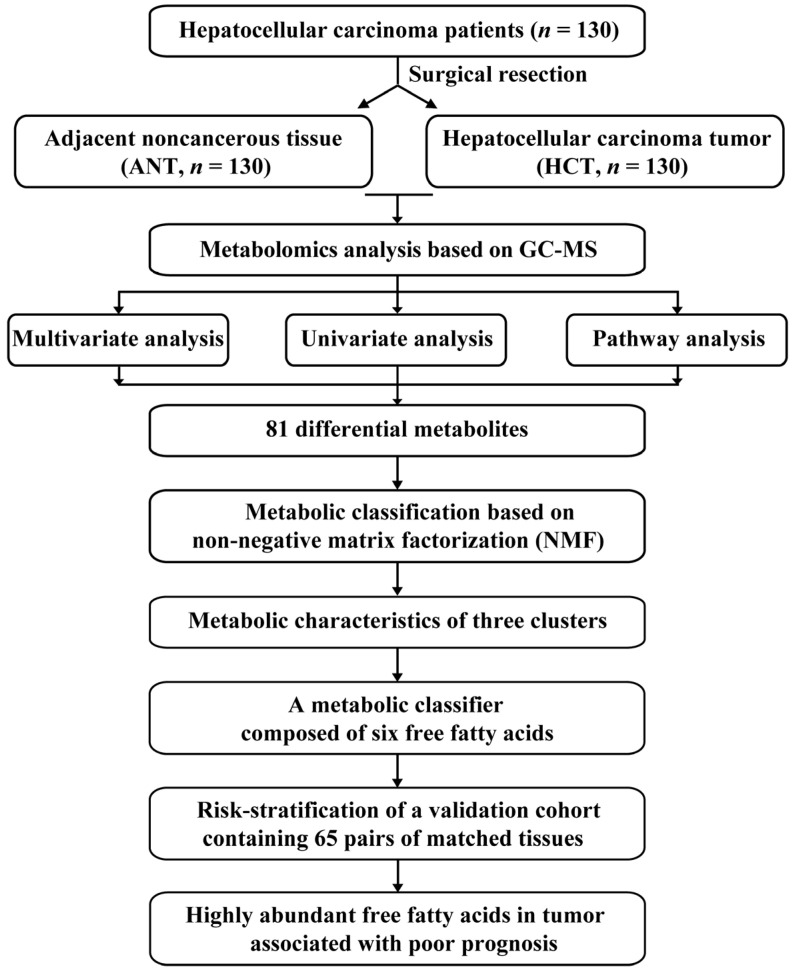
Flow chart of analysis process.

In this study, a metabolic profiling method based on gas chromatography–mass spectrometry (GC-MS) was applied to explore the metabolic reprogramming of 130 pairs of matched tumor tissues (hepatocellular carcinoma tissue (HCT) and adjacent noncancerous tissue (ANT). Nonnegative matrix factorization (NMF) clustering [19,20] was carried out to define different metabolic clusters based on differential metabolites and further to identify a metabolite classifier that could enable HCC prognosis risk stratification. Another batch composed of 65 pairs of matched tissues was enrolled as an external validation to further study the relationship between metabolic clusters and clinical prognosis. The flow chart of the analysis process is shown in Figure 1.

## 2. Materials and Methods

### 2.1. Chemicals

Methanol (high-performance liquid chromatography grade) was purchased from Merck (Darmstadt, Germany). Ultrapure water was filtered through a Milli-Q water system (EMD Millipore Corporation, Billerica, MA, USA). Derivatization reagents including methoxyamine hydrochloride, pyridine and N-methyl-N-(trimethylsilyl)-trifluoroacetamide (MSTFA) were supplied by Sigma-Aldrich (St. Louis, MO, USA). Valine-d8, succinic acid-d4, phenylalanine-d5, tridecanoic acid, citric acid-d4 and myristic acid-d27 were used as internal standards and obtained from Sigma-Aldrich. Metabolite standards for structure identification were acquired from Sigma-Aldrich, Alfa Aesar (Ward Hill, MA, USA), Fluka (Seelze, Niedersachsen, Germany) and J&K Scientific (Beijing, China).

### 2.2. Patients and Tumor Samples

Matched pairs of HCT and ANT samples were obtained from 130 patients undergoing curative resection of HCC at the Eastern Hepatobiliary Surgery Institute of the Second Military Medical University from July 2013 to June 2014. The ANTs were collected from the adjacent edge, less than 2 cm away from the solid tumor border. Another batch of 65 matched pairs of HCT and ANT samples collected from The First Affiliated Hospital of Dalian Medical University were used as an external validation cohort. All samples were freshly frozen and stored at −80 °C prior to metabolomics analysis.

Patients were followed up after surgical resection, and overall survival was defined as the time from the date of surgery to the time of death. For the subjects who still survived at the end of the follow-up period, the latest follow-up time was counted as the endpoint of overall survival. During the follow up, 42 of the patients from the Eastern Hepatobiliary Surgery Hospital of the Second Military Medical University survived, and 38 of them died (the other patients were lost to follow up). Among the patients from The First Affiliated Hospital of Dalian Medical University, 51 of the patients survived, and 14 of them passed away. The detailed clinical characteristics of all patients are listed in Table 1. Tumor stages were defined according to the eighth edition of tumor-node-metastasis (TNM) classification (the American Joint Committee on Cancer/Union for International Cancer Control), and the Barcelona Clinic Liver Cancer (BCLC) staging system.

### 2.3. Sample Preparation and Metabolic Profiling Analysis

A piece of tissue (~10 mg) was mixed with 600 μL of cold 80% methanol solution containing 5 μg/mL internal standards (valine-d8, succinic acid-d4, phenylalanine-d5, tridecanoic acid, citric acid-d4 and myristic acid-d27) and then homogenized using a high-speed blender after vortexing for 30 s. The mixture was centrifuged at 4 °C (14,000× *g*) for 10 min to remove the protein fraction. Finally, 480 μL of the supernatant was lyophilized in Labconco vacuum concentrators and then subjected to an oximation reaction with 50 μL methoxyamine–pyridine solution (20 mg/mL) at 37 °C for 1.5 h, and a silylation reaction with 40 μL of MSTFA at 37 °C for 1 h for subsequent metabolic profiling analysis. Quality control (QC) samples, which were technical replicates by pooling an equal aliquot of extracts from all experimental samples, were processed with the same method as the experimental samples and inserted every 10 samples during the analytical batch to monitor the data quality.

Metabolic profiling analysis was performed on a TQ 8050 NX GC-MS system in single-quadrupole full-scan mode coupled with AOC-20i automatic sampler (Shimadzu, Japan), and a DB-5MS fused-silica capillary column (30 m  ×  0.25 mm  ×  0.25 µm, Agilent Technologies, Santa Clara, CA, USA) was used. The column temperature was maintained at 80 °C for the first 1 min, increased to 210 °C at 30 °C/min, and then ramped to 320 °C at a rate of 20 °C/min, and ultimately kept for 4 min. The injection volume was 1 μL with a 40:1 split ratio (50:1 in the external validation) and the detector voltage was set at 1.11 kV (1.18 kV in the external validation). Other detailed system parameters of the metabolomics method were published in our previous studies [21].

The peaks in a QC sample were deconvoluted by importing the NetCDF file into ChromTOF 4.43 software (Leco Co., St. Joseph, MI, USA) and then identified by comparing the derivatized mass fragments and retention index based on commercial standard libraries (NIST, Gaithersburg, MD, USA, Wiley, Hoboken, NJ, USA; FiehnLib, Davis, CA, USA, and Mainlib, Morris, MN, USA) and a house-made metabolite library.

### 2.4. Statistical Analyses

Before statistical analysis, all metabolic features were calibrated to internal standards with the RSD-minimum principle and tissue weight [22]. QCs and samples were unsupervised clustered by principal component analysis (PCA) to observe the degree of clustering, and the global alteration of metabolic profiling between the ANT group and the HCT group was monitored by supervised partial least squares discriminant analysis (PLS-DA) by SIMCA-P program (version 11.0, Umetrics, Umeå, Sweden). A permutation test was applied to assess the reliability of the multivariate model and avoid overfitting. To explore the metabolic variation, paired (Wilcoxon test) and nonpaired (Mann–Whitney U-test) nonparametric tests were used to identify the significantly differential metabolites between two groups, and the Kruskal–Wallis test was used for comparison among the three clusters using SPSS Statistics software (version 25.0, IBM SPSS, Chicago, IL, USA). False discovery rates (FDRs) were also calculated to the correct *p* value using the Benjamini–Hochberg method through the MATLAB program (MathWorks, Portola Valley, CA, USA). Hierarchical cluster analysis (HCA) was performed using the MultiExperiment Viewer software package (MeV, version 4.7.1) (http://mev.tm4.org (accessed on 31 December 2021)), and the relative responses of differential metabolites were plotted in histograms using the GraphPad software (version 5.01, La Jolla, CA, USA). Metabolic clustering was run by a flexible R package named NMF [19] based on NMF algorithms [20] for 200 iterations of best rank, with default settings of method brunet and seed random. Rank survey was completed using 200 iterations of ranks 2–10, the value of k when the magnitude of the cophenetic correlation coefficient began to fall was chosen as the optimal number of clusters [23]. Volcano plots were drawn to visualize the metabolic differences between groups or clusters. Metabolic clustering and volcano plots were implemented by R 4.0.2 version. Kaplan–Meier curves and log-rank test results were generated using MedCalc Software (version 19.0.4, MedCalc Software, Ostend, Belgium). Cox regression analyses of metabolic cluster and clinicopathological parameters associated with overall survival were completed by using SPSS Statistics software (version 25.0, IBM SPSS, USA).

## 3. Results

### 3.1. Metabolic Profiling Analysis of HCC Tissues

To enable comprehensive metabolic disturbance in HCC, a nontargeted GC-MS-based metabolomics strategy was applied to obtain metabolic profiling in 130 pairs of matched fresh HCT and ANT samples, encompassing tumors of different clinical TNM and BCLC staging systems (Table 1). A total of 135 metabolic characteristic ions from 115 metabolites were quantified and corrected by internal standards and tissue weight. In QC samples, 94.8% of the metabolites had RSD values less than 30%, and the corresponding area accounted for 92.2% of total peak area (Figure S1a). The PCA score scatter plot based on metabolites with RSD value less than 30% was constructed, which showed that the tumor tissues were clearly separated from the noncancerous tissues, and QC samples were tightly clustered (Figure S1b). Therefore, the metabolomics method was sufficiently stable and reliable, and the data of metabolic profiling was well measured. Metabolites with RSD% value less than 30% in QC samples were retained for further analysis.

### 3.2. Metabolic Reprogramming of HCC

**Figure 2:**
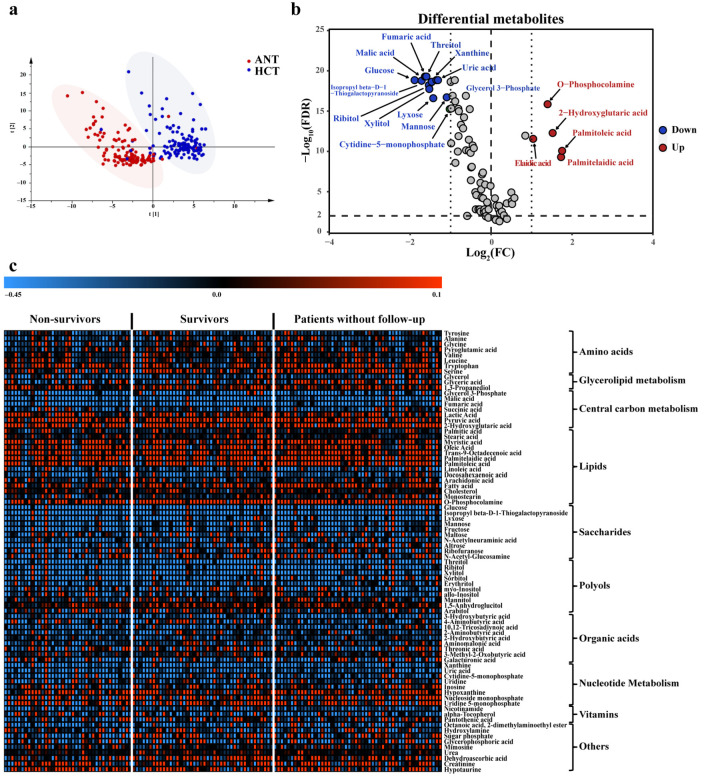
Metabolic signatures of the adjacent noncancerous tissue (ANT) and hepatocellular carcinoma tumor (HCT) group. (**a**) Partial least squares discriminant analysis (PLS-DA) score scatter plot of ANT and HCT groups. (**b**) Volcano plot of the 81 significant differential metabolites (FDR < 0.05) between ANT and HCT groups. Paired nonparametric test (Wilcoxon test) was used to calculate statistical significance, and *p* values were corrected using the Benjamini–Hochberg method. FDR, false discovery rate. FC, fold change. (**c**) Heatmap of 81 metabolites with significant changes by comparing HCT group with ANT group. Red, increased metabolite. Blue, decreased metabolite. The red arrows represent significant upregulation in the HCT group.

As shown in the PLS-DA score plot, the HCT group was clearly separated from the ANT group on the first principal component (Figure 2a), and no over-fitting was observed. The result of running the permutation test 200 times showed the intercept values of R2Y and Q2Y were 0.097 and −0.270, respectively (Figure S1c), meaning there was no overfitting. After FDR correction, 81 metabolites (21 higher and 60 lower in HCT) were identified as differential metabolites by paired nonparametric tests (Wilcoxon test), which displayed differential abundance between HCT and ANT samples (FDR corrected *p* value < 0.05) (Tables S1 and S2, and Figure 2b). Among them, palmitoleic acid, palmitelaidic acid, 2-hydroxyglutaric acid, O-phosphocolamine and elaidic acid were most abundant in tumors (fold change > 2), while glucose, malic acid, fumaric acid and 10 other metabolites (threitol, ribitol, isopropyl beta-D-1-thiogalactopyranoside, xylitol, glycerol 3-phosphate, lyxose, xanthine, uric acid, mannose and cytidine-5-monophosphate) were notably downregulated (fold change < 0.5).

To visualize the variation trends of differential metabolites, the logarithms of the fold changes (HCT/ANT) to the base 10 were plotted in a heatmap (Figure 2c), and the metabolites were clustered according to their types. Overall, significant differences between the HCT group and ANT group existed in amino acids, central carbon metabolism-related metabolites, lipids, saccharides, polyols, organic acids, nucleotides, vitamins and other compounds. The levels of tumor-dependent serine and tryptophan were significantly increased in the HCT group, which have been reported to become therapeutic targets for tumor targeted therapy [24,25]. Consistent with the Warburg effect [26], most of the metabolites involved in the tricarboxylic acid (TCA) cycle were decreased, including succinic acid, fumaric acid and malic acid, while pyruvate and lactic acid were significantly increased corresponding to highly activated glycolysis. An obvious increase was observed in the levels of monounsaturated fatty acids (MUFAs), whereas the polyunsaturated fatty acid (PUFA) levels decreased, which confirmed our previous findings [12]. It was noteworthy that all detected saccharides and most of the polyols had a low abundance in tumor tissues (e.g., glucose, mannose, threitol, ribitol), which may be associated with the abnormally activated glucose metabolism and greatly decreased fructose metabolism in malignant tumors, representing the impact of cancerization on energy metabolism. In addition, hypoxanthine presented an elevated trend in the HCT group, whereas xanthine declined drastically, which was consistent with the prior metabolomics analysis of paired HCC tissues [12].

**Figure 3:**
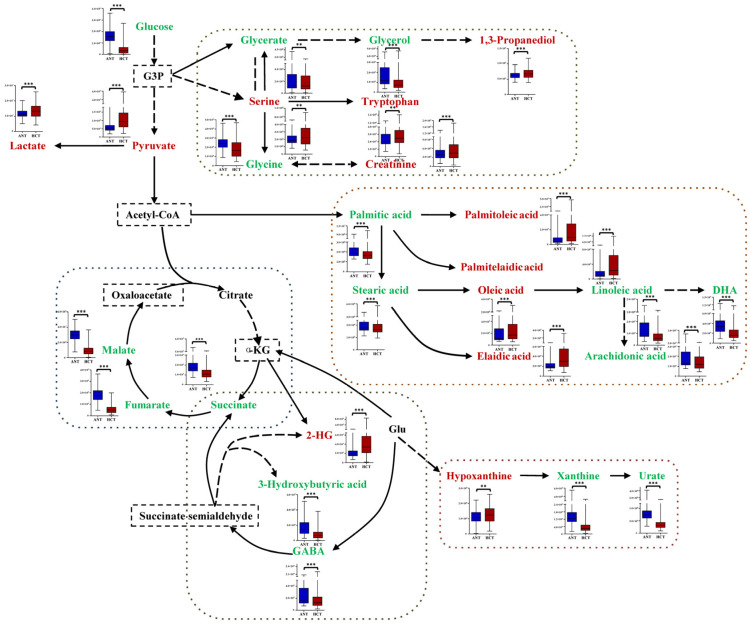
Metabolic pathways of some significantly changed metabolites in HCC. The blue and red bars represent the corrected responses in the ANT and HCT groups, respectively. The colors of names represent changes in HCT group compared with ANT group. Red, increased metabolite. Green, decreased metabolite. Dark, not significantly changed. Dotted box, not measured. Dotted arrow, indirect link. ** and *** represent *p* value of less than 0.01 and 0.001, respectively. Others the same as Figure 2.

To investigate the metabolic reprogramming, a metabolic pathway map to depict changes between the two groups was constructed based on the differential metabolites (Figure 3). Elevated levels of pyruvate and lactate, the major glycolytic products, were observed in the HCT group, indicating an enhanced global glycolysis flux. Moreover, three intermediate products (succinic acid, fumaric acid and malic acid) in the TCA cycle were markedly reduced, which suggested a low level of ATP production through aerobic phosphorylation. As sources of one carbon unit, serine and tryptophan showed increased abundance to accelerate fueling on synthesis of purine and pyrimidine. Due to the vigorous catabolism and high energy demand in cancer cells, FFA metabolism played a pivotal role in tumor metabolic reprogramming as well. Interestingly, the content of PUFA was obviously decreased contrary to the increase of MUFA, which may suggest inhibition of the desaturation pathway and the promotion of β-oxidation [12]. Hypoxanthine was oxidized to xanthine under the catalysis of xanthine oxidoreductase (XOR), and then converted to uric acid subsequently. Accumulation of hypoxanthine and the reduction of downstream metabolites may be connected to the inhibited activity of XOR in HCC that blocked the purine catabolism.

### 3.3. HCC Risk Stratification Based on Metabolic Phenotypes

**Figure 4:**
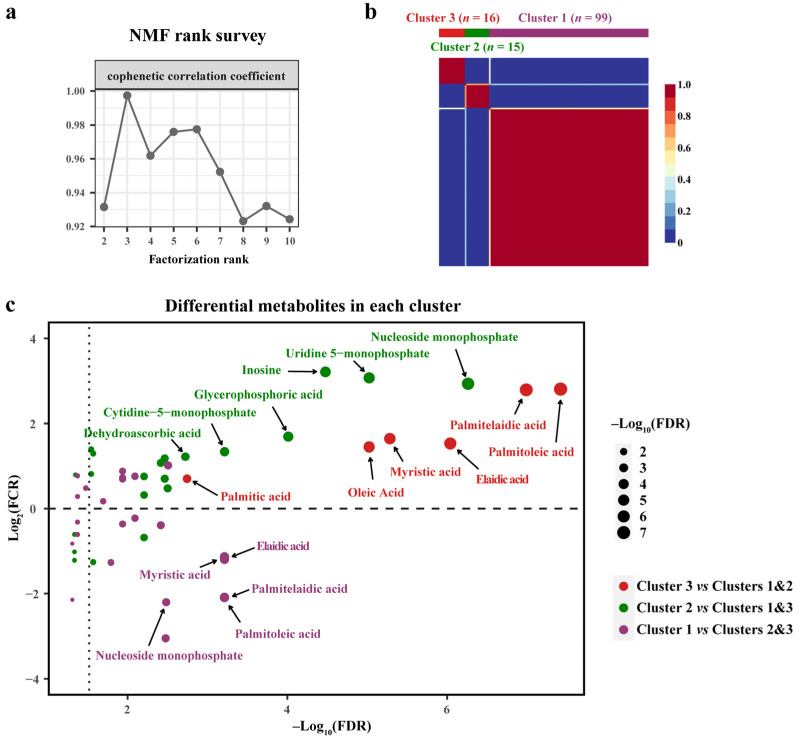
Unsupervised clustering of HCC based on metabolic signatures in the discovery cohort. (**a**) Relationship between rank and cophenetic correlation coefficient after NMF rank survey. (**b**) NMF clustering based on fold change of differential metabolites between ANT and HCT groups. (**c**) Volcano plot shows which differential metabolites have a significantly increased or decreased fold change in each cluster, relative to all other clusters. NMF, nonnegative matrix factorization. FDR, false discovery rate. FCR, fold change (HCT/ANT) ratio. Others the same as Figure 2.

To evaluate whether HCC tumors could be clustered according to their own distinct metabolic phenotypes, unsupervised metabolic clustering was performed by applying NMF consensus clustering. The fold changes of differential metabolites in the HCT compared to the ANT groups were subjected to clustering. Cophenetic correlation coefficients were calculated after NMF rank survey, and k = 3 was determined as the optimal number of clusters (Figure 4a). Three metabolic clusters were yielded as shown in the consensus matrix heatmap (Figure 4b). To better understand the metabolic characteristics of the three HCC clusters, the Mann–Whitney U-tests were used to determine which metabolites were significantly altered in each cluster relative to the remainder of the cohort (FDR corrected *p* value < 0.05). For each cluster, the log10-(FDR-corrected *p* value) of significantly changed metabolites was plotted as the *X*-axis, and the log2-(fold change ratio) of these metabolites was plotted as the *Y*-axis. Interestingly, Cluster 3 was characterized by the highest fold change of six FFAs (palmitoleic acid, palmitelaidic acid, elaidic acid, myristic acid, oleic acid and palmitic acid) in the HCT to the ANT, while Cluster 1 had the most significant low fold change of four FFAs (palmitoleic acid, palmitelaidic acid, myristic acid, elaidic acid), and Cluster 2 mainly displayed a higher fold change of nucleotides (Figure 4c). Meanwhile, nonparametric test results showed that increasing and decreasing trends of metabolites in each cluster were basically consistent (Table S3).

**Figure 5:**
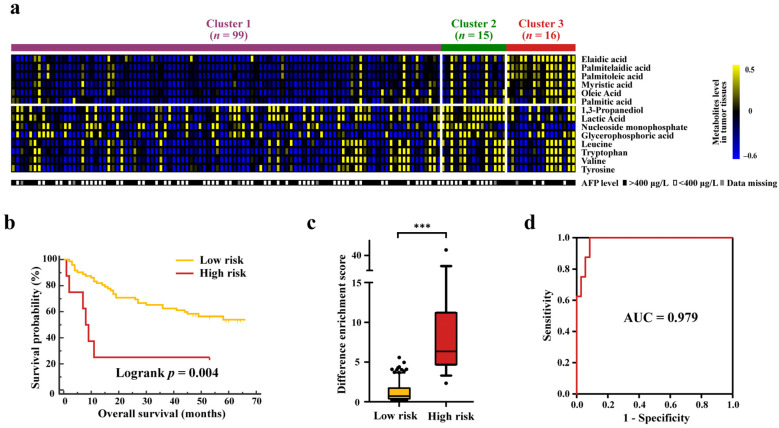
Association between metabolic clusters and prognosis of HCC in the discovery cohort. (**a**) Heatmap of the differential metabolites in tumor tissues and AFP among three clusters. The comparison among three clusters was completed by Kruskal–Wallis test. (**b**) Kaplan–Meier curves of overall survival of low-risk group (Clusters 1 and 2) and high-risk group (Cluster 3). (**c**) DES of low-risk and high-risk groups in the discovery cohort. Mann Whitney test was used to calculate *p* value, and *** represent *p* value of less than 0.001. The black dots are not in the 90% confidence interval. (**d**) ROC curve of DES for prognosis prediction. DES, difference enrichment score.

To further characterize the HCC metabolic clusters, differential metabolites in tumor tissues among the three clusters were defined by performing the Kruskal–Wallis test. A total of 14 differential metabolites had discrepant levels after FDR correction, of which the same 6 FFAs (elaidic acid, palmitelaidic acid, palmitoleic acid, myristic acid, oleic acid and palmitic acid, *p* value from low to high) showed the most striking differences in tumor tissues. Notably, Cluster 3 still had significantly higher levels of six FFAs than Clusters 1 and 2, but different from the ratio analysis of HCT to ANT, Cluster 2 exhibited higher levels of eight other metabolites (1,3-propanediol, lactic acid, nucleoside monophosphate, glycerophosphoric acid, leucine, tryptophan, valine and tyrosine, *p* value from low to high) (Figure 5a).

In addition to significant differences in metabolic characteristics, the alpha fetoprotein (AFP) level was also distinct among the three clusters. The proportion of patients with higher AFP level (>400 μg/L) in Cluster 3 accounted for 80%, which was significantly higher than that in the other clusters, and this was consistent with the poor prognosis of Cluster 3 (Figure 5a, Table S4). The overall survival of patients in each cluster was assessed, and it was found that Cluster 3 with higher levels of FFAs exhibited the worst clinical outcome among three clusters (log-rank test, *p* = 0.016) (Figure S2a). Three clusters were classified into low-risk (Clusters 1 and 2) and high-risk (Clusters 3) groups of mortality, and they were clearly stratified as well (log-rank test, *p* = 0.004) (Figure 5b). Univariate and multivariable Cox regression analyses with overall survival were performed, and risk stratification defined by metabolic clustering was shown to be an independent prognostic factor for HCC, indicating an association between metabolic characteristics and prognosis (Table 2). In addition, TNM stage was also a significant prognostic risk predictor, and the high-risk group had the highest proportion (44%) of advanced stage (III) tumors (Figure S2b).

### 3.4. Validation of Highly Abundant Free Fatty Acids’ Association with Poor HCC Prognosis

**Figure 6:**
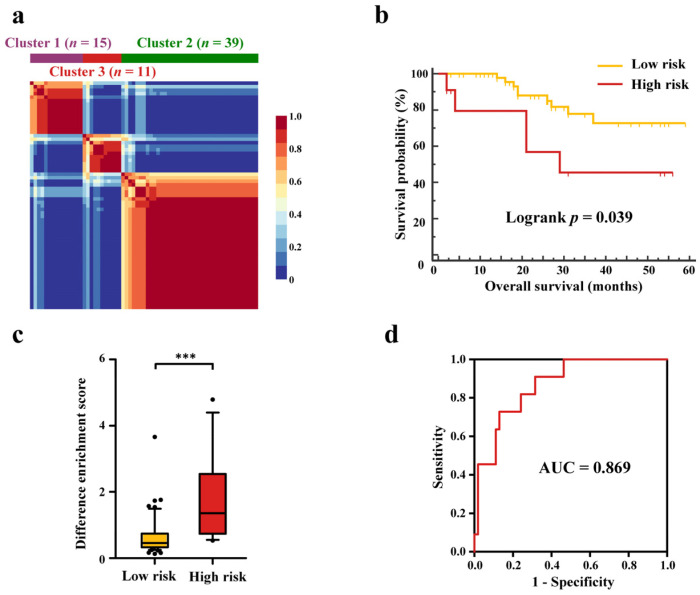
Unsupervised clustering of HCC based on metabolic signatures of six free fatty acids in the validation cohort. (**a**) NMF clustering based on fold change of six FFAs between ANT and HCT group. (**b**) Kaplan–Meier curves of overall survival of low-risk group (Clusters 1 and 2) and high-risk group (Cluster 3). (**c**) DES of low-risk and high-risk groups. Mann Whitney test was used to calculate *p* value, and *** represent *p* value of less than 0.001. The black dots are not in the 90% confidence interval. (**d**) ROC curve of DES for prognosis prediction. Others the same as Figure 2 and Figure 5.

Unsupervised metabolic clustering identified one cluster of high-risk patients whose tumors were characterized by high levels of FFAs (Cluster 3) (Figure 4 and Figure 5). To validate the correlation between metabolic characteristics and prognosis, another dataset of tissue metabolomics was acquired and divided into three clusters as well by subjecting the fold changes of six FFAs to NMF clustering (Figure 6a). Results of Mann–Whitney U-tests showed that Cluster 3 exhibited a significantly increased fold change in three FFAs (palmitoleic acid, oleic acid and myristic acid), relative to Clusters 1 and 2 (FDR corrected *p* < 0.05), and there was also an upward trend in three other FFAs (Figure S3a). Then, the overall survival of patients was examined, and results showed that the high-risk group (Clusters 3) exhibited worse clinical outcome than the low-risk group (Clusters 1 and 2) (log-rank test, *p* = 0.039) (Figure 6b and Figur S3b). The above results verified that HCC patients could be classified based on the metabolite classifier composed of six FFAs, and patients with higher levels of these metabolites in tumors may have a worse prognosis.

A difference enrichment score (DES) was defined to quantify and evaluate the differences in six FFAs (palmitoleic acid, palmitelaidic acid, elaidic acid, myristic acid, oleic acid and palmitic acid) between HCT and ANT samples in each patient.

(1) DES=∑i=1n=6(cFFAiHCT−cFFAiANTcFFAiANT)26,

A significant difference was observed in DES between low-risk and high-risk groups, with higher DES of Cluster 3 than those of Clusters 1 and 2 (*p* < 0.001; Figure 5c), which indicated that DES might be useful for prognostic prediction. The receiver operating characteristic (ROC) curve of DES was obtained, and the area under the curve (AUC) was 0.979 for discriminating low-risk and high-risk groups in the discovery cohort (Figure 5d). Based on the average DES of the low-risk group (Clusters 1 and 2), the cutoff value for HCC prognosis risk assessment was determined as 1.27 with 100.0% sensitivity and 68.1% specificity. Meanwhile, the Mann–Whitney U-test showed that DES of the validation cohort was also significantly different between two risk groups (*p* < 0.001; Figure 6c). Furthermore, the sensitivity and specificity for identifying high-risk patients from the validation cohort were 63.6% and 88.9%, respectively, with an AUC value of 0.869 (Figure 6d).

## 4. Discussion

In recent years, the study of cancer metabolism has attracted great interest due to the identification of tumorigenic mutations in metabolic genes and the potential discovery of drug targets from the metabolism characteristics. Accurate early diagnosis and prognosis evaluation of cancer can be achieved through the detection of various biomarkers. Most of the metabolic markers are found in biological fluid samples, which help to diagnose malignant tumors by noninvasive means. However, it is limited to the determination of organ-specific markers only by biological fluid samples, because they are easily affected by other organs and systemic environment. In order to provide a systematic insight into the metabolism of HCC, a nontargeted metabolomics method was used to analyze the metabolic profiling of paired HCT and ANT samples. Tissue metabolomics comparing tumor tissues with matched adjacent noncancerous tissues could eliminate as many individual differences as possible, such as age, gender, region, etc.

### 4.1. Characteristics of Metabolic Reprogramming in HCC Tissue

Significant metabolic disturbances occurred during the process of tumorigenesis and development, such as enhanced glycolysis, reduced TCA cycle and upregulated β-oxidation. Additionally, metabolic reprogramming occurs in nucleotides and other metabolites to adapt to tumor proliferation and invasion.

Adequate energy supply is essential for the growth and proliferation of tumors. According to the Warburg effect, even in the presence of sufficient oxygen, cancer cells prefer to activate aerobic glycolysis rather than rely on oxidative phosphorylation. The levels of glucose, malic acid and other intermediate metabolites in the TCA cycle were significantly downregulated, while those of pyruvate and lactic acid were significantly upregulated in the HCT group, which revealed that cancer cells consumed glucose rapidly to produce energy through glycolysis, while energy acquisition through the TCA cycle was inhibited. This may be in part due to the dysfunction of mitochondria, or the change of enzymes in cancer cells, especially the altered activity of three key glycolytic enzymes (hexokinase, phosphofructokinase and pyruvate kinase) [27,28,29]. Furthermore, the glycolytic pathway produces ATP faster than the TCA cycle, and provides NADPH to maintain the internal redox homeostasis, as well as provides substrates for the synthesis of biological macromolecules [30,31,32]. In addition to glucose catabolism, β-oxidation in mitochondria is another important source to produce a large amount of energy and NADPH [33], which is regarded to be upregulated in HCC by extended PPARα activation [34], corresponding to the increased contents of MUFAs. Previous studies confirmed that the ratio of acetylcarnitine to carnitine (C2/C0) was increased in the HCT group, suggesting that the β-oxidation of even-numbered FFAs was upregulated [12]. In addition, the level of PUFA reduced significantly in HCT group, while PUFA has been shown to exhibit anti-inflammatory and anti-oxidant properties and to restrain the production of pro-inflammatory cytokines [35,36]. In particular, docosahexaenoic acid, the omega-3 PUFA, has also been reported to reduce the HCC cell growth through inhibition of the signal transduction of prostaglandin E2 by downregulating COX-2 and upregulating 15-hydroxyprostaglandin dehydrogenase, a COX-2 antagonist [37].

Amino acids play an important role in cell metabolism. As a nonessential amino acid, serine is an intermediate connecting saccharide, lipid and nucleotide metabolism, and it produces a marked effect in maintaining tumor proliferation and homeostasis [38]. Serine contributing methyl to ensure the one carbon cycle and generate NADPH for anti-oxidant defense was shown to be upregulated in tumor tissues in our results. A large amount of serine is needed to maintain cell viability, and exogenous serine starvation stress can inhibit the proliferation of a variety of cancer cells [24,39]. In vivo, serine is synthesized through the serine synthesis pathway, from the intermediate product of glycolysis, 3-phosphoglycerate. It has been found that phosphoglycerate dehydrogenase (PHGDH), the rate-limiting step of serine synthesis, is overexpressed in triple-negative breast cancer, while the knockdown of PHGDH significantly affects cell growth [40,41]. In addition to the expression level, the enzyme activity of PHGDH in rectal cancer cells and sarcoma models also increased significantly [42], and it has become a new target for cancer treatment and drug discovery at present. The degradation of glycine provides 5,10-methylenetetrahydrofolate, which is an intermediate product of the folate cycle, and inhibition of glycine decarboxylase in the degradation leads to the disruption of cell homeostasis [42]. Although the content of glycine decreased in tumor tissues in our results, it did not affect cancer cell proliferation because serine is an important source of glycine synthesis. However, if serine is deficient and the synthesis of serine is blocked, the growth of cells will be significantly inhibited [39].

The activity of DNA and RNA polymerases in tumor tissues was higher than that in adjacent noncancerous tissues. Correspondingly, the process of nucleotide catabolism was significantly reduced. XOR, a key enzyme catalyzing the decomposition of hypoxanthine, had a significantly lower expression level in HCC tissues. The activity and expression of XOR were confirmed to be abnormal in various tumors and related to the prognosis [43,44], suggesting that XOR may be involved in tumorigenesis through different molecular mechanisms. Recently, XOR was found to aggravate the accumulation of ROS in liver cancer stem cells by promoting ubiquitin-specific peptidase 15 (USP15)-mediated nuclear factor erythroid 2-related factor 2 (Nrf2)-Kelch-like ECH associated protein 1 (KEAP1) signaling, ultimately block liver cancer stem cell and tumor propagation [45]. In summary, cancer cells obtained energy and substrates needed for survival and proliferation by adjusting their metabolic pathways, meanwhile reducing apoptosis by counteracting oxidative stress.

### 4.2. Metabolite Classifier for HCC Risk Stratification

The metabolic abnormalities and phenotypes of HCC are highly heterogeneous, which suggests that the influence of heterogeneity should not be ignored in the study [46]. With the expanding of the understanding of cancer molecular typing, some genome- and transcriptome-based molecular classifications of HCC have been revealed in recent years [18,47]. Previous joint studies of genomics and metabolomics highlighted the heterogeneity of gene expression and metabolic alterations in the same pathway, and the importance of generating a complete atlas by combining the two data types was highlighted [48]. Metabolic risk stratification based on the characteristics of the metabolome could enrich the molecular pathological information of tumorigenesis and progression, and provide assistance for clinically individualized therapeutics and prognosis evaluation [10].

Notably, three metabolic clusters were identified by unsupervised clustering analysis, and high levels of FFAs in tumor tissues were the key characteristics of the cluster with poor prognosis, accompanied by a high level of AFP. The positive correlation between abundance of the FFAs and prognosis was further validated by another metabolomics dataset. The FFAs that composed the metabolite classifier were either saturated or monounsaturated and have been reported to get involved in the formation and prognosis of HCC. Upregulated oxidation of fatty acids, as a driving force for tumor proliferation, may also be closely related to the HCC prognosis. As confirmed, MUFAs produced by stearoyl-CoA desaturase provided a Wnt-positive signaling loop via stabilization of low-density lipoprotein-receptor-related proteins 5 and 6, which contributed to liver fibrosis and tumor growth [49]. Palmitic acid increased the level of acetylated AFP by disrupting SIRT1-mediated deacetylation, which was associated with poor prognosis and decreased patient survival [50]. In addition, the growth inhibition caused by knockdown of lipolytic enzyme acyl-CoA thioesterase 8 could be partially rescued by the addition of myristic acid in HCC [51]. The indicated importance of FFA metabolism was helpful to improve the understanding of the pathogenesis of HCC. Nevertheless, the specific molecular mechanism of HCC still needs to be elucidated thoroughly in the future.

NMF can effectively reduce the dimension of a large-scale matrix, extract and classify features, indicating the correlation between information, which has great potential in academic research. The risk stratification defined by NMF unsupervised clustering was an independent prognostic factor for HCC, revealing an association between metabolic characteristics and prognosis. ROC analysis results showed that the classifier constructed by the pivotal metabolic characteristics of the high-risk group could well distinguish the patients with different levels of HCC prognostic risk. Hence, metabolic classification based on the metabolic characteristics of HCC tissues in this study offered a new insight for the heterogeneity of HCC and could be used as a new method to explore the molecular features of tumors and prognosis.

Clinically, surgical resection is one of the optimal options for patients in early stage, but for patients who have not received surgical resection or are on drug therapy, the prognosis cannot be evaluated based on tissue samples. Therefore, it is still necessary to integrate widely accepted clinical parameters and biomarkers for a comprehensive prognostic risk assessment.

## 5. Conclusions

Overall, our study demonstrated that nontargeted metabolomics strategy was a reliable tool to explore the metabolic characteristics of HCC. HCC was characterized by distinct metabolic reprogramming to satisfy energy and substance demand for tumor cell survival and proliferation. Briefly, the Warburg effect that cancer cells were more inclined to rely on active aerobic glycolysis was verified in our study, and the TCA cycle showed an obvious downregulation. Moreover, β-oxidation of FFAs was upregulated to obtain energy and reductants, while all detected saccharides and most of the polyols had a low abundance in tumor tissues due to the abnormal activation of carbohydrate metabolism. Three metabolic clusters with different characteristics were classified by applying NMF consensus clustering based on the ratio analysis of HCT to ANT. The cluster characterized by the highest fold change of FFAs exhibited a worst prognosis, which was further validated in another dataset of tissue metabolomics. Risk-stratification based on a classifier composed of six FFAs may provide new insights into the clinical prognosis evaluation and personalized treatment of HCC.

## Figures and Tables

**Table 1:** Clinical characteristics of patients ^a,b^.

Characteristics	Discovery Cohort (*n* = 130)				Validation Cohort (*n* = 65)		
	All (*n* = 130)	Patients with Follow Up (*n* = 80)	Survivors (*n* = 42)	Nonsurvivors (*n* = 38)	All (*n* = 65)	Survivors (*n* = 51)	Nonsurvivors (*n* = 14)
Age, years	49 (10.9)	49 (10.9)	49 (11.0)	50 (11.0)	60 (9.8)	60 (10.2)	62 (8.4)
Gender, Male/Female	114 (87.7)/16 (12.3)	69 (86.3)/11 (13.8)	36 (85.7)/6 (14.3)	33 (86.8)/5 (13.2)	55 (84.6)/10 (15.4)	44 (86.3)/7 (13.7)	11 (78.6)/3 (21.4)
Smoking, Yes/No	61 (46.9)/69 (53.1)	36 (45.0)/44 (55.0)	19 (45.2)/23 (54.8)	17 (44.7)/21 (55.3)	34 (52.3)/31 (47.7)	27 (52.9)/24 (47.1)	7 (50.0)/7 (50.0)
Hepatitis B, Positive/Negative	103 (79.2)/27 (20.8)	62 (77.5)/18 (22.5)	37 (92.5)/3 (7.5)	31 (81.6)/7 (18.4)	48 (73.8)/17 (26.2)	37 (72.5)/14 (27.5)	11 (78.6)/3 (21.4)
Cirrhosis, Presence/Absence	25 (19.4)/104 (80.6), *n* = 129	12 (15.0)/68 (85.0)	2 (4.8)/40 (95.2)	10 (26.3)/28 (73.7)	45 (69.2)/20 (30.8)	33 (64.7)/18 (35.3)	12 (85.7)/2 (14.3)
AFP level, μg/L, >400/<400	58 (46.8)/66 (53.2), *n* = 124	38 (48.7)/40 (51.3), *n* = 78	18 (45.0)/22 (55.0), *n* = 40	20 (52.6)/18 (47.4)	15 (23.4)/49 (76.6), *n* = 64	10 (19.6)/41 (80.4)	5 (38.5)/8 (61.5), *n* = 13
ALP level, U/L	90 (36–911), *n* = 121	86 (36–911), *n* = 77	74 (36–735), *n* = 40	98 (50–911), *n* = 37	98 (51–312)	89 (51–312)	113.5 (65–221)
GGT level, U/L	62 (13–525), *n* = 121	62 (14–438), *n* = 77	43 (14–438), *n* = 40	111 (15–297), *n* = 37	67 (14–384)	64 (14–384)	77.5 (20–213)
Bilirubin, μmol/L	13.7 (5.5–32.7), *n* = 124	13.9 (6.7–32.7), *n* = 78	13.6 (6.7–32), *n* = 40	14.6 (6.9–32.7)	14 (7.0–93.0)	14.1 (7.8–93.0)	12.9 (7.0–33.2)
Albumin, g/L	42.0 (3.5), *n* = 124	42.0 (3.7), *n* = 78	42.0 (3.0), *n* = 40	42.0 (4.3)	40.6 (5.1)	41 (4.5)	38 (6.3)
TNM stage, Stage I/Stage II/Stage III	57 (43.8)/32 (24.6)/41 (31.5)	37 (46.3)/17 (21.3)/26 (32.5)	27 (64.3)/10 (23.8)/5 (11.9)	10 (26.3)/7 (18.4)/21 (55.3)	39 (60.0)/20 (30.8)/6 (9.2)	32 (62.7)/16 (31.4)/3 (5.9)	7 (50.0)/4 (28.6)/3 (21.4)
BCLC stage, Stage 0/Stage A/Stage B/Stage C	2 (1.5)/82 (63.1)/15 (11.5)/31 (23.8)	1 (1.3)/53 (66.3)/7 (8.8)/19 (23.8)	1 (2.4)/36 (85.7)/0 (0.0)/5 (11.9)	0 (0.0)/17 (44.7)/7 (18.4)/14 (36.8)	0 (0.0)/42 (64.6)/5 (7.7)/18 (27.7)	0 (0.0)/34 (66.7)/3 (5.9)/14 (27.5)	0 (0.0)/8 (57.1)/2 (14.3)/4 (28.6)
Maximum tumor diameter, cm	7.4 (1.3–17.8)	7.1 (1.7–17.8)	4.7 (1.7–17.8)	9.2 (3.2–17.2)	4.3 (0.3–14.5)	4 (1.5–12.0)	5 (0.3–14.5)
Tumor number, ≥2/1	20 (15.4)/110 (84.6)	11 (13.8)/69 (86.3)	0 (0.0)/42 (100.0)	11 (28.9)/27 (71.1)	10 (15.6)/55 (85.9)	6 (11.8)/45 (88.2)	4 (28.6)/10 (71.4)
Microvascular invasion, Presence/Absence	52 (40.0)/78 (60.0)	33 (41.3)/47 (58.8)	13 (31.0)/29 (69.0)	20 (52.6)/18 (47.4)	10 (15.6)/55 (85.9)	8 (15.7)/43 (84.3)	2 (14.3)/12 (85.7)

^a^ All parameters were detected before surgery. Age and albumin were expressed as average (SD). ALP level, GGT level, bilirubin and maximum tumor diameter were expressed as median (range). Other characteristics were expressed as number (proportion%). ^b^ *n* is as indicated in the column headings unless otherwise state. AFP, alpha fetoprotein; ALP, alkaline phosphatase; GGT, gamma-glutamyl transferase; SD, standard deviation.

**Table 2:** Univariate and multivariate Cox regression analyses of metabolic cluster and clinicopathological parameters associated with overall survival ^a^.

Variable	Overall Survival (*n* = 80)			
	Univariate		Multivariate	
	HR (95% CI)	*p* Value	HR (95% CI)	*p* Value
Metabolic risk stratification ^b^, High risk/Low risk	3.38 (1.40, 8.18)	0.007	3.16 (1.27, 7.87)	0.013
Cirrhosis, Presence/Absence	3.37 (1.62, 7.02)	0.001	-	-
Maximum tumor diameter, cm	1.09 (1.02, 1.16)	0.009	-	-
Tumor number, ≥2/1	5.48 (2.58, 11.62)	9.00 × 10−^6^	-	-
Microvascular invasion, Presence/Absence	1.96 (1.04, 3.72)	0.038	-	-
TNM Stage ^c^	-	2.29 × 10−^5^	-	0.014
TNM Stage II	1.65 (0.63, 4.34)	0.309	1.55 (0.59, 4.08)	0.377
TNM Stage III	5.50 (2.56, 11.81)	1.25 × 10−^5^	3.89 (1.61, 9.42)	0.003
BCLC Stage ^d^	-	2.08 × 10−^5^	-	-
BCLC Stage B	6.82 (2.73, 17.06)	4.07 × 10−^5^	-	-
BCLC Stage C	4.01 (1.96, 8.19)	1.39 × 10−^4^	-	-

^a^ HR, hazard ratio; CI, confidence interval. ^b^ Clusters 1 and 2 were merged as low-risk group. ^c^ Stage I was used as the reference group. ^d^ Stage 0 + A was used as the reference group.

## Data Availability

The data presented in this study are available on request from the corresponding author.

## Supplementary Materials

The following are available online at https://www.mdpi.com/article/10.3390/cancers14010231/s1. Table S1. The derivatized mass fragments and retention index of the differential metabolites. Table S2. Fold change (HCT/ANT), *p* value and FDR value of detected metabolites. Table S3. Alteration of metabolites in each cluster by comparing HCT group with ANT group. Table S4. Clinical alpha fetoprotein level of three metabolic clusters. Figure S1. Evaluation of the data quality. (a) RSD distribution of peaks in QC samples. (b) PCA score scatter plot of samples and QCs. (c) Permutation plot for PLS-DA Model. Figure S2. The prognosis of metabolic clusters in the discovery cohort, Figure S3: Association between metabolic clusters and prognosis of HCC in the validation cohort.

## Abbreviations

Hepatocellular carcinoma (HCC), gas chromatography–mass spectrometry (GC-MS), hepatocellular carcinoma tissue (HCT), adjacent noncancerous tissue (ANT), free fatty acids (FFAs), N-methyl-N-(trimethylsilyl)-trifluoroacetamide (MSTFA), tumor-node-metastasis (TNM), Barcelona Clinic Liver Cancer (BCLC), quality control (QC), Automated Mass Spectral Deconvolution and Identification system (AMDIS), principal components analysis (PCA), partial least squares discriminant analysis (PLS-DA), false discovery rates (FDRs), hierarchical cluster analysis (HCA), nonnegative matrix factorization (NMF), relative standard deviation (RSD), monounsaturated fatty acids (MUFAs), polyunsaturated fatty acids (PUFAs), saturated fatty acids (SFAs), xanthine oxidoreductase (XOR), alpha fetoprotein (AFP), tricarboxylic acid (TCA), receiving operator characteristic (ROC), area under the curve (AUC), phosphoglycerate dehydrogenase (PHGDH), ubiquitin-specific peptidase 15 (USP15), nuclear factor erythroid 2-related factor 2 (Nrf2), Kelch-like ECH-associated protein 1 (KEAP1).
