# Supplementary material for: Metabolic Reprogramming and Risk Stratification of Hepatocellular Carcinoma Studied by Using Gas Chromatography–Mass Spectrometry-Based Metabolomics
Source: Cancers (Basel). 2022 Jan 4;14(1):231. doi: 10.3390/cancers14010231 (PMC8750553; doi:10.3390/cancers14010231)
Supplement: Supplementary file 1 [file cancers-14-00231-s001.zip › cancers-1501620-supplementary.pdf]

cancers

MDPI

Supplementary Material

Metabolic Reprogramming and Risk-Stratification of Hepatocellular Carcinoma Studied by Using Gas Chromatography - Mass Spectrometry-Based Metabolomics

Chengnan Fang [1,2], Hui Wang [3], Zhikun Lin [4], Xinyu Liu [1], Liwei Dong [3], Tianyi Jiang [3], Yexiong Tan [3], Zhen Ning [4], Yaorui Ye [1], Guang Tan [4,*] and Guowang Xu[1,*]

[1] CAS Key Laboratory of Separation Science for Analytical Chemistry, Dalian Institute of Chemical Physics, Chinese Academy of Sciences, Dalian 116023, China; chengnanf@dicp.ac.cn (C.N.F.); liuxy2010@dicp.ac.cn (X.Y.L.); g1808@dicp.ac.cn (Y.R.Y.); xugw@dicp.ac.cn (G.W.X.)
[2] University of Chinese Academy of Sciences, Beijing 100049, China
[3] International Cooperation Laboratory on Signal Transduction, Eastern Hepatobiliary Surgery Institute, The Second Military Medical University, Shanghai 200438, China; 13816444416@163.com (H.W.); dlw@smmu.edu.cn (L.W.D.); 08300700057@fudan.edu.cn (T.Y.J.); yxtan1214@163.com (Y.X.T.)
[4] Department of Hepatobiliary Surgery, The First Affiliated Hospital of Dalian Medical University, Dalian 116011, China; linzk@dicp.ac.cn (Z.K.L.); ningzhen@dmu.edu.cn (Z.N.); guangtan@dmu.edu.cn (G.T.)
* Correspondence: guangtan@dmu.edu.cn (G.T.); xugw@dicp.ac.cn (G.W.X.)

Table S1. Derivatized mass fragments and retention index of the differential metabolites.

Name	Retention Index	Unique mass
1,2-Propanediol	990	117
Ethanolamine 1	1023	102
2-Aminobutyric acid	1025	58
2-Hydroxypyridine	1034	152
Pyruvic acid	1039	174
1,3-Propanediol	1049	130
Lactic Acid	1050	117
Valine 1	1085	72
Alanine 1	1094	116
Hydroxylamine	1113	133
Glycine 1	1113	102
2-Hydroxybutyric acid	1116	131
1-Piperidinecarboxaldehyde	1134	98
3-Hydroxybutyric acid	1150	191
Leucine 1	1153	86
Isoleucine 1	1175	86
Proline 1	1178	70
Mimosine	1186	188
Valine 2	1207	144
Urea	1232	189
Serine	1249	132
Glycerol	1259	205
Ethanolamine 2	1261	174
Leucine 2	1262	158
Isoleucine 2	1283	158
Threonine 1	1285	130
Proline 2	1293	142
3-Methyl-2-Oxobutyric acid	1300	103
Glycine 2	1300	174
Succinic acid	1301	247
Glyceric acid	1314	292
Uracil	1329	241
Fumaric acid	1338	245
Nonanoic acid	1354	215
Alanine 2	1356	188
Threonine 2	1371	218
Methionine	1410	104
Aspartic acid	1414	160
Aminomalonic acid	1457	218
Trans-4-Hydroxyproline	1466	158

D-Threitol	1470	217
Malic acid	1470	233
Nicotinamide	1482	179
Erythritol	1486	217
Pyroglutamic acid 1	1497	84
Pyroglutamic acid 2	1516	156
Trans-4-Hydroxyproline	1520	230
4-Aminobutyric acid	1520	174
Glutamic acid 1	1520	84
2,3,4-Trihydroxybutyric acid	1522	292
Threonic acid	1537	292
Creatinine	1551	329
Phenylalanine 1	1553	120
2-Hydroxyglutaric acid	1557	247
Phosphoenolpyruvic acid	1581	369
Asparagine	1587	159
Hypotaurine	1598	188
Glutamic acid 2	1602	246
Phenylalanine 2	1625	192
D-lyxose	1655	103
Xylitol	1685	307
Arabitol	1699	217
Ribitol	1705	217
Dihydroxyacetone phosphate	1723	400
Glycerol 3-Phosphate	1738	357
D-Ribofuranose	1747	217
2-Phosphoglycericacid	1755	369
O-Phosphocolamine	1771	188
3-Phosphoglyceric acid	1784	357
Hypoxanthine	1802	265
1,5-Anhydroglucitol	1837	217
Dehydroascorbic acid 1	1839	173
Myristic acid	1841	285
Tagatose	1850	307
D-Fructose	1866	103
Tyrosine 1	1885	179
D-Mannose	1887	103
D-Glucose	1901	103
Dehydroascorbic acid 2	1901	244
D-Mannitol	1912	319
D-Sorbitol	1921	319
Tyrosine 2	1930	218

Isopropyl beta-D-1-Thiogalactopyranoside	1939	217
Galacturonic acid	1952	333
Pantothenic acid	1982	291
Mucic acid	2001	333
Xanthine	2004	353
Cytidine-5-monophosphate	2004	243
Allo-inositol	2013	318
Palmitoleic acid	2018	311
Palmitelaidic acid	2019	311
Palmitic acid	2038	313
N-Acetyl-D-Glucosamine	2062	202
Uric acid 1	2080	441
Myo-Inositol	2081	305
D-Altrose 1	2117	319
D-Altrose 2	2124	319
Uric acid 2	2137	369
Linoleic acid	2207	337
Tryptophan 1	2211	202
Oleic Acid	2212	339
Trans-9-Octadecenoic acid	2219	339
Glycerophosphoric acid	2224	357
Stearic acid	2236	117
Tryptophan 2	2236	202
Octanoic acid, 2-dimethylaminoethyl ester	2282	58
D-Fructose-6-Phosphate	2284	315
D-Glucose-6-phosphate	2298	387
Sugar phosphate 1	2336	315
Arachidonic acid	2367	80
Fatty acid	2388	80
D-Myo-Inositol 4-Monophosphate	2403	318
Uridine	2444	217
Sugar phosphate 2	2466	387
Sugar phosphate 3	2533	387
Inosine	2557	230
10,12-Tricosadiynoic acid	2557	79
Docosahexaenoic acid	2559	91
Glycerol 1-Palmitate	2572	371
N-Acetylneuraminic acid	2611	246
Maltose	2723	361
Monostearin	2765	399
Nucleoside monophosphate	2814	169
Uridine 5-monophosphate	2871	169

Alpha-Tocopherol	3145	502
Cholesterol	3177	329

Table S2. Fold change (HCT/ANT), *p* value and FDR value of detected metabolites.

Name	*p* value	FDR value	Fold change (HCT/ANT)
Malic acid	7.97×10^{-22}	5.20×10^{-20}	0.32
Threitol	9.54×10^{-22}	5.20×10^{-20}	0.33
Fumaric acid	1.46×10^{-21}	5.31×10^{-20}	0.32
Glucose	9.42×10^{-21}	1.47×10^{-19}	0.27
Xanthine	7.08×10^{-21}	1.47×10^{-19}	0.39
Uric acid	7.90×10^{-21}	1.47×10^{-19}	0.40
Nicotinamide	8.26×10^{-21}	1.47×10^{-19}	0.54
Isopropyl beta-D-1-Thiogalactopyranoside	1.28×10^{-20}	1.74×10^{-19}	0.30
Glycerol 3-Phosphate	1.98×10^{-20}	2.36×10^{-19}	0.36
3-Hydroxybutyric acid	2.16×10^{-20}	2.36×10^{-19}	0.50
Ribitol	6.23×10^{-20}	6.18×10^{-19}	0.34
Xylitol	1.97×10^{-19}	1.79×10^{-18}	0.35
Octanoic acid, 2-dimethylaminoethyl ester	1.60×10^{-18}	1.25×10^{-17}	0.52
2-Hydroxybutyric acid	1.60×10^{-18}	1.25×10^{-17}	0.65
Mannose	2.62×10^{-18}	1.91×10^{-17}	0.47
Lyxose	3.64×10^{-18}	2.48×10^{-17}	0.37
Erythritol	9.47×10^{-18}	6.07×10^{-17}	0.54
O-Phosphocolamine	2.29×10^{-17}	1.39×10^{-16}	2.63
Fructose	8.13×10^{-17}	4.67×10^{-16}	0.51
10,12-Tricosadiynoic acid	9.52×10^{-17}	5.19×10^{-16}	0.57
Cytidine-5-monophosphate	1.07×10^{-16}	5.55×10^{-16}	0.49
Tyrosine	1.16×10^{-16}	5.73×10^{-16}	0.69
Docosahexaenoic acid	2.20×10^{-16}	1.04×10^{-15}	0.58
2-Aminobutyric acid	1.32×10^{-15}	6.01×10^{-15}	0.63
Glycerol	1.18×10^{-14}	5.14×10^{-14}	0.60
Succinic acid	3.31×10^{-14}	1.39×10^{-13}	0.62
2-Hydroxyglutaric acid	1.27×10^{-13}	5.14×10^{-13}	2.87
Pyruvic acid	2.83×10^{-13}	1.10×10^{-12}	1.80
Linoleic acid	3.36×10^{-13}	1.26×10^{-12}	0.57
Trans-9-Octadecenoic acid	7.51×10^{-13}	2.73×10^{-12}	2.06
N-Acetylneuraminic acid	1.00×10^{-12}	3.52×10^{-12}	0.68
Sorbitol	2.92×10^{-12}	9.95×10^{-12}	0.50
Glycine	5.54×10^{-12}	1.83×10^{-11}	0.73
Aminomalonic acid	1.23×10^{-11}	3.93×10^{-11}	0.74
Myo-Inositol	2.32×10^{-11}	7.24×10^{-11}	0.65

Uridine	2.85×10^{-11}	8.54×10^{-11}	0.68
Palmitoleic acid	2.90×10^{-11}	8.54×10^{-11}	3.38
Maltose	3.62×10^{-11}	1.04×10^{-10}	0.57
Alanine	5.43×10^{-11}	1.52×10^{-10}	0.70
Palmitelaidic acid	1.89×10^{-10}	5.14×10^{-10}	3.32
4-Aminobutyric acid	1.01×10^{-9}	2.69×10^{-9}	0.54
Allo-inositol	3.23×10^{-9}	8.37×10^{-9}	0.72
Altrose	3.99×10^{-9}	1.01×10^{-8}	0.71
Alpha-Tocopherol	1.72×10^{-8}	4.25×10^{-8}	0.87
3-Methyl-2-Oxobutyric acid	2.93×10^{-8}	7.10×10^{-8}	0.86
Hydroxylamine	1.60×10^{-7}	3.78×10^{-7}	0.71
Mannitol	3.41×10^{-7}	7.90×10^{-7}	0.83
Palmitic acid	5.68×10^{-7}	1.29×10^{-6}	0.90
Galacturonic acid	8.43×10^{-7}	1.88×10^{-6}	0.99
Monostearin	2.41×10^{-6}	5.25×10^{-6}	0.84
Ribofuranose	5.40×10^{-6}	1.15×10^{-5}	0.76
Myristic acid	5.90×10^{-6}	1.24×10^{-5}	1.41
Inosine	1.27×10^{-5}	2.61×10^{-5}	0.78
Arachidonic acid	2.64×10^{-5}	5.33×10^{-5}	0.85
Hypotaurine	3.02×10^{-5}	5.98×10^{-5}	1.49
1,5-Anhydroglucitol	3.41×10^{-5}	6.64×10^{-5}	1.07
Mimosine	3.48×10^{-5}	6.65×10^{-5}	0.87
Oleic Acid	8.23×10^{-5}	1.55×10^{-4}	1.43
Stearic acid	1.14×10^{-4}	2.11×10^{-4}	0.93
Fatty acid	1.57×10^{-4}	2.86×10^{-4}	1.31
Uridine 5-monophosphate	1.93×10^{-4}	3.44×10^{-4}	1.43
1,3-Propanediol	3.06×10^{-4}	5.39×10^{-4}	1.10
Lactic Acid	3.29×10^{-4}	5.69×10^{-4}	1.15
N-Acetyl-Glucosamine	8.16×10^{-4}	0.002	0.77
Glycerophosphoric acid	7.44×10^{-4}	0.002	0.83
Pyroglutamic acid	8.37×10^{-4}	0.002	0.89
Urea	6.44×10^{-4}	0.002	0.96
Sugar phosphate 3	0.001	0.002	0.81
Tryptophan	0.001	0.002	1.14
Threonic acid	0.002	0.003	0.83
Pantothenic acid	0.002	0.003	0.88
Valine	0.002	0.003	0.91
Serine	0.002	0.003	1.20
Hypoxanthine	0.003	0.004	1.16
Creatinine	0.006	0.009	1.24
Glyceric acid	0.007	0.010	0.66
Leucine	0.008	0.011	0.92

Cholesterol	0.012	0.017	0.95
Nucleoside monophosphate	0.017	0.023	1.31
Arabitol	0.023	0.031	1.08
Dehydroascorbic acid	0.035	0.047	1.15
Aspartic acid	0.043	0.056	0.94
1,2-Propanediol	0.043	0.056	0.97
2,3,4-Trihydroxybutyric acid	0.050	0.065	1.21
Phenylalanine	0.066	0.085	1.09
Threonine	0.073	0.093	0.92
Asparagine	0.090	0.113	0.93
Myo-Inositol 4-Monophosphate	0.116	0.144	1.06
2-Hydroxypyridine	0.125	0.153	1.10
Ethanolamine	0.141	0.171	0.87
Mucic acid	0.151	0.181	1.56
Proline	0.153	0.181	1.05
Methionine	0.168	0.197	0.89
Dihydroxyacetone phosphate	0.185	0.215	0.92
Glutamic acid	0.219	0.251	1.09
Fructose-6-Phosphate	0.265	0.301	1.25
1-Piperidinecarboxaldehyde	0.283	0.318	0.95
Sugar phosphate 1	0.405	0.450	1.01
Phosphoenolpyruvic acid	0.427	0.470	1.21
Tagatose	0.441	0.481	1.11
Nonanoic acid	0.507	0.542	1.03
Glucose-6-phosphate	0.503	0.542	1.27
Glycerol 1-Palmitate	0.581	0.615	1.13
Isoleucine	0.589	0.617	0.97
3-Phosphoglyceric acid	0.623	0.647	1.11
Sugar phosphate 2	0.633	0.651	1.08
Uracil	0.751	0.765	1.02
2-Phosphoglycericacid	0.824	0.832	1.04
Trans-4-Hydroxyproline	0.953	0.953	1.02

Table S3. Alteration of metabolites in each cluster by comparing HCT group with ANT group.

Metabolites	Cluster 1		Cluster 2		Cluster 3	
	Fold change (HCT/ANT)	*p* value	Fold change (HCT/ANT)	*p* value	Fold change (HCT/ANT)	*p* value
Malic acid	0.36	1.21×10^{-16}	0.26	8.05×10^{-4}	0.19	4.38×10^{-4}
Threitol	0.37	1.52×10^{-16}	0.27	8.05×10^{-4}	0.19	4.38×10^{-4}
Fumaric acid	0.36	1.87×10^{-16}	0.25	9.87×10^{-4}	0.20	4.38×10^{-4}
Xanthine	0.39	2.36×10^{-16}	0.45	0.005	0.33	4.38×10^{-4}
Uric acid	0.46	1.76×10^{-16}	0.28	0.003	0.35	4.38×10^{-4}
Nicotinamide	0.56	1.48×10^{-15}	0.54	6.55×10^{-4}	0.43	4.38×10^{-4}
Glucose	0.26	8.73×10^{-17}	0.48	0.020	0.19	5.31×10^{-4}
Isopropyl beta-D-1-Thiogalacto-pyranoside	0.29	8.23×10^{-17}	0.51	0.027	0.23	5.31×10^{-4}
Glycerol 3-Phosphate	0.35	1.17×10^{-16}	0.70	0.125	0.26	6.43×10^{-4}
3-Hydroxybutyric acid	0.51	3.25×10^{-15}	0.47	6.55×10^{-4}	0.47	4.38×10^{-4}
Ribitol	0.33	1.93×10^{-16}	0.52	0.023	0.26	4.38×10^{-4}
Xylitol	0.32	8.87×10^{-16}	0.71	0.112	0.29	4.38×10^{-4}
2-Hydroxybutyric acid	0.66	1.17×10^{-13}	0.64	0.001	0.59	4.38×10^{-4}
Octanoic acid, 2-dimethylami-noethyl ester	0.50	3.07×10^{-15}	0.72	0.069	0.43	6.43×10^{-4}
Mannose	0.45	7.89×10^{-15}	0.67	0.053	0.41	4.38×10^{-4}
Lyxose	0.37	1.89×10^{-14}	0.43	0.015	0.29	4.38×10^{-4}
Erythritol	0.49	1.85×10^{-15}	0.97	0.125	0.45	0.001
O-Phosphocolamine	2.57	7.36×10^{-13}	3.04	6.55×10^{-4}	2.63	0.004
Fructose	0.48	9.21×10^{-14}	0.70	0.112	0.44	6.43×10^{-4}
10,12-Tricosadiynoic acid	0.59	7.25×10^{-14}	0.50	0.017	0.57	0.003
Cytidine-5-monophosphate	0.47	7.67×10^{-15}	1.13	1.000	0.32	4.38×10^{-4}
Tyrosine	0.67	9.99×10^{-13}	0.72	0.005	0.78	0.002
Docosahexaenoic acid	0.60	3.32×10^{-13}	0.46	0.008	0.57	0.006
2-Aminobutyric acid	0.65	1.65×10^{-12}	0.63	0.004	0.52	0.007
Glycerol	0.58	2.29×10^{-12}	0.61	0.005	0.69	0.034
Succinic acid	0.58	2.25×10^{-13}	1.09	1.000	0.49	0.002
2-Hydroxyglutaric acid	2.88	4.46×10^{-10}	3.40	0.003	2.33	0.008
Pyruvic acid	1.72	3.57×10^{-11}	2.02	0.100	2.00	0.005
Linoleic acid	0.58	6.26×10^{-11}	0.45	0.004	0.71	0.070
Trans-9-Octadecenoic acid	1.58	1.30×10^{-8}	2.10	0.031	4.71	4.38×10^{-4}
N-Acetylneuraminic acid	0.71	3.23×10^{-9}	0.71	0.017	0.52	7.76×10^{-4}
Sorbitol	0.42	7.68×10^{-12}	1.17	0.955	0.41	0.001
Glycine	0.73	1.60×10^{-8}	0.79	0.023	0.64	6.43×10^{-4}
Aminomalonic acid	0.72	3.73×10^{-10}	0.95	0.460	0.67	0.003
Myo-Inositol	0.71	8.15×10^{-7}	0.48	0.002	0.44	4.38×10^{-4}

Uridine	0.71	5.18×10^{-8}	0.55	0.002	0.63	0.017
Palmitoleic acid	1.92	2.22×10^{-7}	2.35	0.078	16.04	4.38×10^{-4}
Maltose	0.58	1.06×10^{-8}	0.53	0.053	0.57	0.008
Alanine	0.75	2.73×10^{-7}	0.57	0.005	0.59	0.006
Palmitelaidic acid	1.82	1.86×10^{-6}	2.52	0.112	15.21	4.38×10^{-4}
4-Aminobutyric acid	0.71	3.12×10^{-6}	0.28	0.002	0.36	0.010
Allo-inositol	0.75	3.28×10^{-6}	0.70	0.036	0.51	0.001
Altrose	0.70	1.21×10^{-7}	0.92	0.307	0.51	0.006
Alpha-Tocopherol	0.94	3.12×10^{-6}	0.51	0.009	0.75	0.049
3-Methyl-2-Oxobutyric acid	0.86	8.44×10^{-6}	0.91	0.078	0.78	0.004
Hydroxylamine	0.67	1.06×10^{-6}	0.85	0.125	0.87	0.255
Mannitol	0.80	3.23×10^{-7}	0.89	0.363	0.95	0.255
Palmitic acid	0.83	1.41×10^{-8}	0.90	0.069	1.33	0.134
Galacturonic acid	0.98	8.27×10^{-8}	1.36	0.140	0.76	0.163
Monostearin	0.84	1.28×10^{-5}	0.87	0.233	0.82	0.088
Ribofuranose	0.78	4.37×10^{-5}	0.85	0.532	0.60	0.023
Myristic acid	1.03	0.005	1.58	0.307	4.15	4.38×10^{-4}
Inosine	0.72	1.56×10^{-7}	2.10	0.005	0.72	0.026
Arachidonic acid	0.89	0.001	0.56	0.004	1.01	0.605
Hypotaurine	1.58	7.17×10^{-6}	0.98	0.156	1.57	0.134
1,5-Anhydroglucitol	1.08	1.63×10^{-5}	1.11	0.088	0.98	0.469
Mimosine	0.91	0.005	0.87	0.125	0.66	0.001
Oleic Acid	1.12	0.006	1.24	0.776	3.82	4.38×10^{-4}
Stearic acid	0.94	0.003	0.81	0.011	1.01	0.469
Fatty acid	1.21	6.92×10^{-4}	1.56	0.650	1.62	0.017
Uridine 5-monophosphate	1.31	0.003	10.79	6.55×10^{-4}	0.82	0.278
1,3-Propanediol	1.06	0.086	1.33	9.87×10^{-4}	1.14	0.023
Lactic Acid	1.07	0.105	1.56	8.05×10^{-4}	1.25	0.023
Urea	0.99	0.124	0.87	0.015	0.88	0.002
Glycerophosphoric acid	0.69	3.12×10^{-6}	2.30	0.005	0.77	0.234
N-Acetyl-Glucosamine	0.88	0.147	0.55	0.002	0.59	0.026
Pyroglutamic acid	0.91	0.012	0.89	0.256	0.75	0.034
Sugar phosphate 3	0.79	0.003	1.05	0.496	0.74	0.044
Tryptophan	1.03	0.062	1.26	0.088	1.54	0.011
Pantothenic acid	0.95	0.081	0.88	0.256	0.57	0.002
Serine	1.19	0.008	1.26	0.191	1.19	0.278
Threonic acid	0.99	0.222	0.44	0.005	0.55	0.026
Valine	0.89	0.002	0.94	0.427	0.99	0.796
Hypoxanthine	1.14	0.004	1.44	0.394	1.11	0.408
Creatinine	1.28	0.015	1.21	0.256	1.11	0.301
Glyceric acid	0.76	0.040	0.45	0.008	0.78	0.717
Leucine	0.85	2.80×10^{-4}	1.01	0.865	1.13	0.196

Cholesterol	0.95	0.026	0.91	0.015	1.03	0.605
Nucleoside monophosphate	1.15	0.261	8.10	6.55×10^{-4}	0.78	0.438
Arabitol	1.10	0.085	1.39	0.334	0.70	0.002
Dehydroascorbic acid	1.10	0.249	2.09	0.001	0.97	0.679

Table S4. Clinical alpha-fetoprotein level of three metabolic clusters. [a, b.]

	Cluster 1 (*n* = 99)	Cluster 2 (*n* = 15)	Cluster 3 (*n* = 16)	*p* value
Preoperative AFP level, μg/L, >400/<400	42 (44.2)/53 (55.8), *n* = 95	4 (28.6)/10 (71.4), *n* = 14	12 (80.0)/3 (20.0), *n* = 15	0.013

[a] Preoperative AFP level are expressed as number (proportion%). [b] *n* is as indicated in the column headings unless otherwise state.

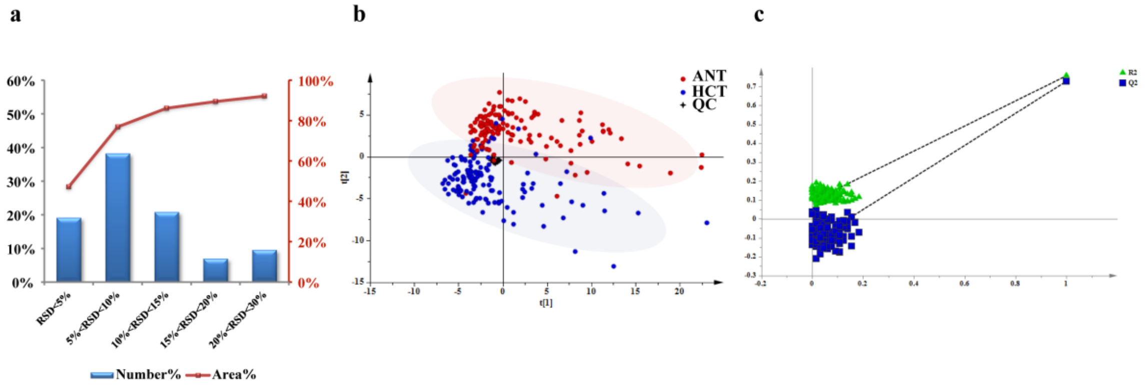

Figure S1. Evaluation of the data quality. **(a)** RSD distribution of peaks in QC samples. **(b)** PCA score scatter plot of samples and QCs. **(c)** Permutation plot for PLS-DA Model.

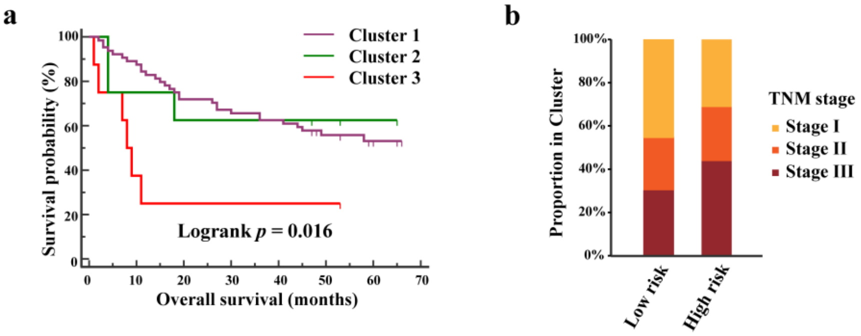

Figure S2. The prognosis of metabolic clusters in the discovery cohort. **(a)** Kaplan-Meier curves of overall survival of each cluster. **(b)** TNM staging of patients in each metabolic cluster.

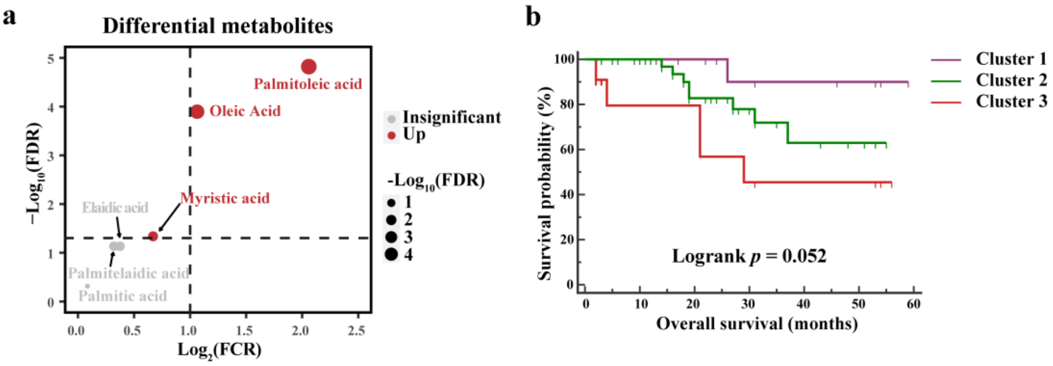

Figure S3. Association between metabolic clusters and prognosis of HCC in the validation cohort. **(a)** Volcano plot shows that three FFAs have a significantly increased fold change in Cluster 3, relative to Clusters 1&2. **(b)** Kaplan-Meier curves of overall survival of each cluster.
